# Spectrum-Effect Relationship between HPLC Fingerprint and Anti-Inflammatory and Analgesic Activities of *Chloranthus fortunei* (A. Gray) Solms-Laub

**DOI:** 10.1155/2023/5697896

**Published:** 2023-07-04

**Authors:** Junhao Shi, Qin Qiu, Xianxing Lu, Dandan Zhen, Xiaofang Liu, Baojun Gu, Chunping Qin, Huiqing Mo, Pengfei Li, Hanshen Zhen

**Affiliations:** ^1^Guangxi University of Chinese Medical, Nanning 530200, China; ^2^Ruikang Hospital Affiliated to Guangxi University of Chinese Medicine, Nanning 530011, China

## Abstract

The predominant objective of the research is to establish the anti-inflammatory and analgesic spectrum-effect relationship of *Chloranthus fortunei* (A. Gray) Solms-Laub (CF), to reveal the pharmacodynamic basis of the anti-inflammatory and analgesic effects of CF. The fingerprints of ten batches of CF from various origins were established by high-performance liquid chromatography (HPLC) and evaluated for similarity, hierarchical cluster analysis (HCA), and principal component analysis (PCA). The anti-inflammatory and analgesic effects of CF were evaluated with the xylene-induced ear swelling in mice and the acetic acid torsion test, while the anti-inflammatory and analgesic spectrum-effect relationship of CF was evaluated by gray relational analysis (GRA) and partial least squares regression analysis (PLSR) to effectively elucidate the anti-inflammatory and analgesic substance basis of CF. The ten batches of CF HPLC fingerprints established in this work successfully identified a total of 13 common peaks that refer to 4 components, with peak 1 being neochlorogenic acid, peak 3 being chlorogenic acid, peak 5 being cryptochlorogenic acid, and peak 10 being rosmarinic acid. The HCA results presented that the ten batches of CF samples were clustered into 3 categories, which was consistent with the PCA results. Simultaneously, the results of the spectrum-effect relationship also indicated that neochlorogenic acid, chlorogenic acid, cryptochlorogenic acid, and rosmarinic acid were the possible anti-inflammatory and analgesic substances of CF. In order to better understand the anti-inflammatory and analgesic substance basis of CF, this experiment established the anti-inflammatory and analgesic spectrum-effect relationship of CF, which can provide a scientific foundation for the quality evaluation and further research as well as the usage of CF herbs.

## 1. Introduction

*Chloranthus fortunei* (A. Gray) Solms-Laub (CF) is a perennial herb which can be mainly discovered in the eastern and southern parts of China. At the same time, it is also a relatively common medicinal herb for the Yao people in China. It possesses the efficacy of dispersing wind and cold, activating blood circulation, relieving pain, detoxifying, and reducing swelling [1, 2]. Concurrently, it has been commonly employed clinically in the treatment of rheumatoid arthritis, bruising, and swelling pain as well as chronic gastroenteritis. At present, the research on this herb has been primarily focused on its chemical composition, but research on the material basis of its anti-inflammatory and analgesic effects has not been comprehensively examined [3, 4].

Inflammation represents a general, complex physiological response of the immune system to external challenges or tissue damage. The occurrence of an inflammatory response assists in the healing of wounds. However, when inflammation is excessive or uncontrollable, an abnormal and prolonged inflammatory response will inevitably result in a variety of diseases [5–7]. Pain has been one of the predominant features of inflammation and is defined by the International Association for the Study of Pain as an unpleasant sensory and emotional experience which has been often associated with actual or potential tissue damage [8–10]. Similarly, there have also been numerous studies that have revealed a definite link between inflammation and pain. As such, the research on the comorbidity of inflammation and pain has unavoidably become increasingly widespread worldwide [11–13].

NSAIDs such as ibuprofen and paracetamol, which have been proven to possess satisfactory anti-inflammatory and analgesic effects and yet also have their own limitations, are the most popular anti-inflammatory and analgesic medications available today [14, 15]. One of their most common shortcomings is gastrointestinal tract injury which is mainly due to the irritation of the drug itself that can cause damage to the gastrointestinal tract which comprises indigestion, nausea and vomiting, and severe pain in the abdomen [16, 17]. As such, this is definitely not what we wish to achieve. Hence, it is necessary to research and develop new highly effective low-toxic anti-inflammatory and analgesic drugs. Simultaneously, the pharmacological basis of its action should also be elucidated.

In recent years, high-performance liquid chromatography (HPLC), which has the advantages of excellent separation efficiency, swift analysis speed, and outstanding detection sensitivity, has been extensively employed in the quality assessment of herbal medicines. This technology allows for quick qualitative and quantitative analysis of the components of the samples analyzed [18–20]. In order to more accurately reflect the complex chemical information contained in traditional Chinese medicine (TCM), fingerprinting technology is utilized in TCM to systematically characterize the chemical components of TCM from the perspective of the overall chemical substance base of TCM, primarily by the HPLC method. By combining fingerprinting with pharmacological effects through relevant statistical methods, the active ingredients of TCM that function can be effectively screened [21, 22]. In the present study, the above methods were organically combined to reveal the anti-inflammatory and analgesic potent substances of CF in a multidimensional manner.

Based on early screening for anti-inflammatory and analgesic efficacy, the aqueous extract of CF with the most optimal anti-inflammatory and analgesic effects was selected for the fingerprinting investigation, and the fingerprinting of the CF aqueous extract was established using the HPLC technique. The fingerprints of the CF aqueous extract from ten different origins were evaluated for similarity, hierarchical cluster analysis (HCA), and principal component analysis (PCA); the anti-inflammatory and analgesic activities of the CF aqueous extract were investigated by constructing a xylene-induced ear swelling model in mice and an acetic acid torsion test in mice. Gray relational analysis (GRA) and partial least squares regression analysis (PLSR) were employed to examine the anti-inflammatory and analgesic spectrum-effect relationships. This study provides a scientific basis for revealing the anti-inflammatory and analgesic substances of CF.

## 2. Materials and Methods

### 2.1. Materials and Reagents

As presented in Table 1, the ten batches of CF herbs had originated from various regions of Guangxi, China, and were verified by experimentalist Zhu Yilin of the Guangxi University of Traditional Chinese Medicine, Guangxi, China. Rosmarinic acid (batch number: RP190718), chlorogenic acid (batch number: RP190505), cryptochlorogenic acid (batch number: RP2101101), and neochlorogenic acid (batch number: RP200416) were purchased from Chengdu Madison Technology Co., Ltd. with purity ≥99.97%; methanol, ethanol, and glacial acetic acid were analytically pure, ultrapure water (laboratory homemade), acetonitrile (chromatographic alcohol, Fisher), and methanol (chromatographic alcohol, Tedia).

### 2.2. Animals

SPF-grade Kunming breed (KM) mice were provided by Hunan Slaughter Jingda Laboratory Animal Co., Ltd., Laboratory Animal Production License No. SCXK (Xiang) 2019-0004. The experimental protocol was approved by the Laboratory Animal Ethics Review Committee of Guangxi University of Traditional Chinese Medicine and was conducted in strict accordance with the relevant regulations, Ethics Review Approval No. DW20201025-137.

### 2.3. Preparation of the Standard Solution and Sample Solution

Chlorogenic acid, neochlorogenic acid, cryptochlorogenic acid, and rosmarinic acid were accurately weighed and dissolved in methanol to prepare mixed standard solutions that contain 219.2 *μ*g/mL of neochlorogenic acid, 282.8 *μ*g/mL of chlorogenic acid, 244 *μ*g/mL of cryptochlorogenic acid, and 343.2 *μ*g/mL of rosmarinic acid.

In the fingerprinting section, 2 g of CF powder was weighed precisely; 25.00 mL of water was added precisely, placed in a flask, weighed and refluxed for 1 h, and then cooled and weighed again to make up the weight. Subsequently, the CF solution to be measured is obtained by thoroughly shaking the mixture, filtering it, and then passing the filtrate through a 0.22 *μ*m microporous membrane.

The CF herbs were utilized in the anti-inflammatory and analgesic experiment in which they were cut into 1 cm pieces, weighed 500 g, added to distilled water (solid-liquid ratio of 1 : 10), and soaked for 30 minutes. They were then twice extracted at reflux for 1.5 hours each time, filtered through medical gauze, combined and concentrated into an infusion, and used after being diluted with distilled water to the corresponding concentration.

### 2.4. Chromatographic Conditions

The column (UltimateXB-C8, 4.6 × 250 mm, and 5 *μ*m) was utilized with methanol (A): 0.1% acetic acid aqueous solution and (B) as the mobile phase for gradient elution: 0∼5 min, 5%∼9% (A); 5∼13 min, 9%∼11% (A); 13∼17 min, 11%∼20% (A); 17∼ 27 min, 20%–23% (A); 27–41 min, 23%–25% (A); 41–47 min, 25%–36% (A); 47–54 min, 36%–43% (A); 54–80 min, 43%–65% (A); detection wavelength 295 nm; volume flow rate 0.8 mL/min; column temperature 30°C; and injection 10 *μ*L.

### 2.5. Validation of Methodology

The sample solution of CF (S1) was accurately prepared in accordance with the sample preparation method in “2.3” for fingerprinting. Next, the sample was injected six times according to the chromatographic conditions in “2.4,” and the precision was checked. Similarly, the stability of the CF (S1) solution was investigated by sample preparation and chromatographic conditions in “2.3” and “2.4” at 0 h, 2 h, 4 h, 8 h, 12 h, and 24 h, respectively. Subsequently, the six replicates of the CF (S1) solution were injected, and the reproducibility of the CF (S1) solution was investigated.

### 2.6. Establishment and Evaluation of Fingerprint

The sample preparation procedure described in “2.3” was implemented to prepare ten batches of CF samples, and the chromatographic conditions described in “2.4” were applied to determine and obtain the relevant profiles. The “Chromatographic Fingerprint Evaluation System for Traditional Chinese Medicine (2012 version)” was utilized to analyze the HPLC fingerprints of the ten batches of CF samples. At the same time, the superimposed spectra and common patterns of the ten batches of CF samples were obtained using the S1 pattern as the reference pattern, and the generated control pattern R was used as the reference. The evaluation of general similarity was performed. Finally, HCA and PCA were applied to the fingerprint profiles of each source [23].

### 2.7. Anti-Inflammatory Test

120 Kunming mice, weighing 18–22 g each, were randomly assigned to 12 groups (*n* = 10, including 5 males and 5 females). Subsequently, ten CF aqueous extract groups of various sources (7.28 g/kg), the positive group (dexamethasone 0.006 g/kg), and the model group (distilled water) were individually set up, and each group shall be administered with 20 mL/kg of the drug once daily for seven days. The mice's right ear was uniformly treated with 20 *μ*L of xylene 45 minutes after the last administration, whereas the left ear was left untreated. After 30 min of inflammation, the mice were executed, the round earpieces were punched with a 6 mm diameter perforator in the same part of both ears and weighed, and the difference in weight between the left and right earpieces was utilized as the degree of swelling. The swelling degree and the swelling inhibition rate were calculated according to the following formula, respectively [24]:

(1) $$\begin{array}{c} swelling\;de\;gree=weight\;of\;the\;right\;ear-weight\;of\;the\;left\;ear\text{.} \end{array}$$

Swelling inhibition rate (%) = (model group ear swelling-administration group ear swelling)/model group ear swelling × 100%.

### 2.8. Analgesic Test

120 Kunming mice, weighing 18–22 g each, were randomly assigned to 12 groups (*n* = 10, including 5 males and 5 females). Each group was set up with the following: the model group (distilled water), the positive group (0.05 g/kg rhododendron), and the aqueous extract group (7.28 g/kg) of ten various sources of CF. Each group shall be administered with 20 mL/kg of the drug once daily for seven days. Simultaneously, 0.6% acetic acid solution (10 mL/kg) was administered intraperitoneally 1 hour after the last administration. The mice torsion times within 15 minutes were counted, and the analgesic inhibition rate was determined using the following formula [25].

Analgesic inhibition rate (%) = (torsion times of the model group-torsion times of administration group)/writhing times of the model group × 100%.

### 2.9. Evaluation of the Anti-Inflammatory Analgesic Spectrum-Effect Relationship

#### 2.9.1. Gray Relational Analysis (GRA)

Gray relation analysis is a research method that aids in decision-making by examining the magnitude of data correlation (the correlation degree between parent sequence and subsequence) and measuring the degree of association between data through correlation. The inhibition rate of mouse ear swelling and the inhibition rate of mouse torsion were taken as the parent sequences, and the peak areas of the common peaks of the fingerprint profiles of ten batches of CF were taken as the subsequences. At the same time, the respective processed data were recorded as *Y*(*k*) and *Xi*(*k*). The raw data were processed and calculated according to formula (2) to successfully obtain the absolute value of the difference between *Y(k)* and *Xi(k)*, respectively. The correlation coefficient is usually utilized to indicate the degree of association between the 2 compared sequences, whereby it can be attained by substituting the absolute value data of the calculated difference series into formula (3), and the discrimination coefficient *ρ* (0∼1) was chosen as 0.5. The correlation degree (*r*) is the degree of association between the two correlations, and the calculated correlation coefficient was brought into formula (4) to calculate the degree of association between each common peak and the anti-inflammatory and analgesic effects of CF [26]. When the correlation degree is greater than 0.6, it indicates that the chemical compounds represented by the chromatographic peaks are correlated with the pharmacological index; when the correlation degree is greater than 0.8, it indicates that the chemical compounds represented by the chromatographic peaks have a great correlation with the pharmacological index; when the correlation degree is greater than 0.9, it indicates that the chemical compounds represented by the chromatographic peaks are very much correlated with the pharmacological index:

(2) $$\begin{array}{c} △\left(ik\right)=\left|Yi\left(k\right)-Xi\left(k\right)\right|\text{,} \end{array}$$

(3) $$\begin{array}{c} \xi i\left(k\right)=\left[\frac{∆\left(\min\right)+\rho ∆\left(\max\right)}{\left(△\left(ik\right)+\rho ∆\left(\max\right)\right.}\right]\text{,} \end{array}$$

(4) $$\begin{array}{c} r=\frac{1.000}{n}\overset{n}{\underset{k=1}{\sum }}\xi i\left(k\right)\text{.} \end{array}$$

#### 2.9.2. Partial Least Squares Regression Analysis (PLSR)

PLSR is a statistical method which combines linear regression analysis, typical correlation analysis, and principal component analysis. By establishing regression equations and comparing regression coefficients and VIP values, the combined contribution of the common peaks to efficacy can be accurately reflected. The trend of the effect of each common peak on efficacy can be determined from the positive and negative values of the regression coefficients, where a positive value means that the common peak is positively correlated with efficacy and a negative value means that it is negatively correlated with efficacy. The variable importance in projection (VIP) of PLSR is an important index reflecting the explanatory ability of the independent variable on the dependent variable, and the larger the value, the stronger the explanatory ability of the independent variable on the dependent variable. It is generally accepted that when VIP > 1, the independent variable has significant importance in explaining the dependent variable. The peak area of each common peak in the fingerprint was utilized as the independent variable *X*. The inhibition rates of CF aqueous extract on ear swelling in mice as well as the inhibition rate of analgesia in mice were utilized as the dependent variables *Y*. Concurrently, the PLSR of the anti-inflammatory and analgesic spectrum-effect relationship of CF was successfully performed using the SIMCA 14.1 software [27].

## 3. Results

### 3.1. Method Validation for HPLC Fingerprint

**Figure 1:**
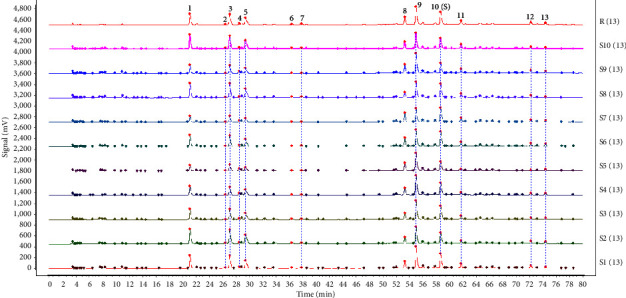
HPLC fingerprints of 10 batches of CF samples.

**Figure 2:**
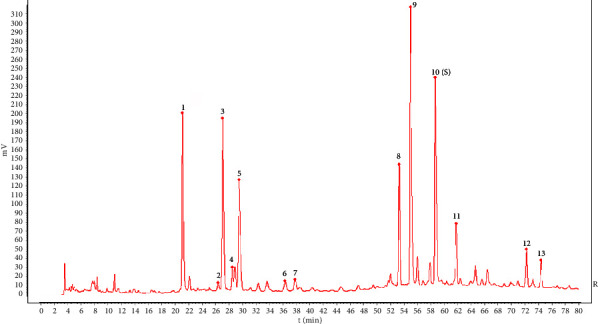
Reference atlas from 10 CF chromatograms.

**Figure 3:**
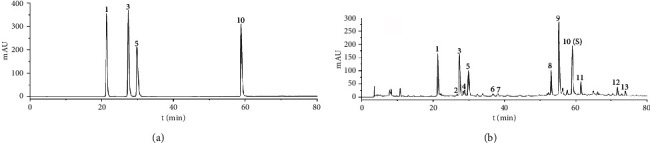
HPLC chromatograms of mixed standard solution (a) and sample (b) (1: neochlorogenic acid, 3: chlorogenic acid, 5: cryptochlorogenic acid, and 10: rosmarinic acid).

Peak 10 (rosmarinic acid), chosen as the reference peak to assess the HPLC fingerprinting methodology, has excellent stability and an appropriate peak area. The results demonstrated that the approach has better precision, stability, and repeatability and is acceptable for CF fingerprinting because the RSDs of the relative retention times and relative peak areas of the other 12 peaks were less than 3%. Figures 1 and 2 display the HPLC fingerprints and reference chromatograms of ten batches of CF samples. 13 peaks that were well separated were designated as common peaks. Four peaks were identified in the fingerprint, with peak 1 being neochlorogenic acid, peak 3 being chlorogenic acid, peak 5 being cryptochlorogenic acid, and peak 10 being rosmarinic acid (Figure 3). Eventually, peak 10 (rosmarinic acid) was selected as the reference peak (*S*), and the RSD of the relative retention time of each common peak in the ten batches of CF samples was calculated to be less than 0.64% (*n* = 10), indicating that the reproducibility among samples was satisfactory; the results have been presented in Table 2. However, the RSD of their relative peak areas ranged from 23.13% to 50.65% (*n* = 10) with large fluctuations, indicating that the content of coconstituents in the samples of CF from different sources varied substantially. The relevant results have been displayed in Table 3.

### 3.2. Similarity Analysis of Fingerprints

The overall similarity was evaluated with the reference fingerprint (*R*) (Table 4). The similarity between the fingerprint profiles of each origin and the reference fingerprint (*R*) was greater than 0.900, indicating that the preparation method of CF was stable and that the deviation between the origins was small. Hence, it was able to satisfy the requirements of fingerprinting studies.

### 3.3. Statistical Analysis of Fingerprints

#### 3.3.1. Hierarchical Cluster Analysis (HCA)

**Figure 4:**
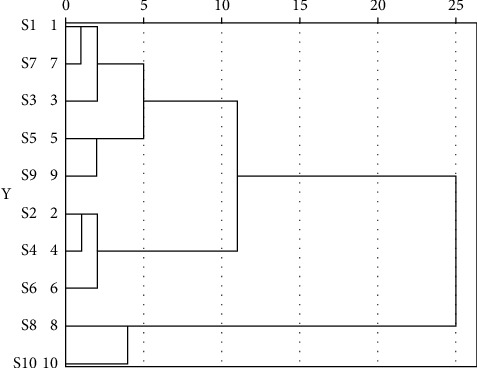
Hierarchical cluster analysis of 10 batches of CF samples.

By utilizing the SPSS26.0 software, the peak areas of 13 common peaks in ten batches of CF samples were employed as variables, and the intergroup linkage clustering method was selected, followed by clustering with the metric of squared Euclidean distance. The results have been presented in Figure 4. When the classification distance was 10, ten batches of CF samples could be classified into three classes. Among them, samples S1, S3, S5, S7, and S9 were clustered into class 1 and samples S2, S4, and S6 were clustered into class 2, while the remaining samples were clustered into class 3. The clustering results reflected that there were certain differences among the ten batches of CF samples, indicating that the differences in environmental factors among different origins might be the predominant influencing factors for their quality differences.

#### 3.3.2. Principal Component Analysis (PCA)

**Figure 5:**
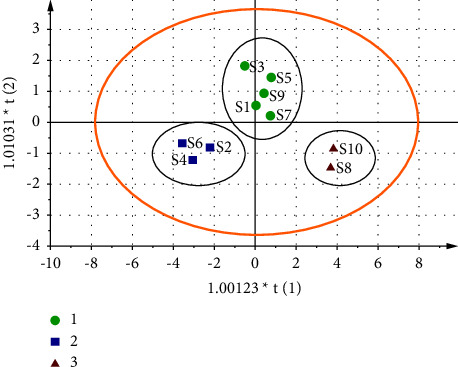
Principal component analysis score of CF samples.

The eigenvalues and variance contribution rates were determined by PCA with SPSS26.0 software using the peak areas of 13 common peaks from ten batches of CF samples as the variables. The outcomes are displayed in Table 5. Using eigenvalues >1 as the criterion, three principal components were obtained with a cumulative variance contribution of 83.40%, representing most of the information of the samples. Therefore, the ten batches of CF samples were evaluated using the first three main components as indicators. According to Table 6, which depicts the common factor loading matrix, the common peaks 1, 2, 3, 5, 6, and 7 have obviously positive loadings in principal component 1, while peaks 4 and 9 have obviously positive loadings in principle component 2. From Figure 5, it can be observed that the ten batches of CF samples can be divided into 3 categories. Among them, samples S1, S3, S5, S7, and S9 were clustered into class 1 and samples S2, S4, and S6 were clustered into class 2, while samples S8 and S10 were clustered into class 3, which was consistent with the results of cluster analysis.

### 3.4. Results of the Anti-Inflammatory Test

**Figure 6:**
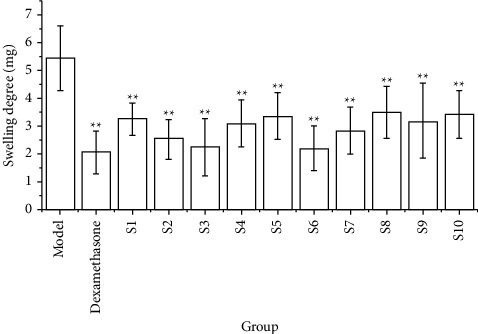
Effects of ten batches of CF aqueous extract on xylene-induced ear swelling in mice (*n* = 10) (compared with the model group ^*∗*^: *P* < 0.05 and ^*∗∗*^: *P* < 0.01).

Compared with the model group, the ear swelling of each origin aqueous extract group was significantly reduced, the difference was statistically significant (*P* < 0.05), and the anti-inflammatory inhibition rate was greater than 35%. Among these, the anti-inflammatory inhibition rate was higher in S2, S3, and S6, and the inhibition rate of each origin aqueous extract group was smaller than that of the positive group (dexamethasone). Therefore, this indicates that the aqueous extract groups of CF will be able to reduce ear swelling in mice and possess a certain inhibitory effect on xylene-induced inflammation. However, their anti-inflammatory effect is relatively weaker than that of the positive group, see Table 7 and Figure 6.

### 3.5. Results of the Analgesic Test

**Figure 7:**
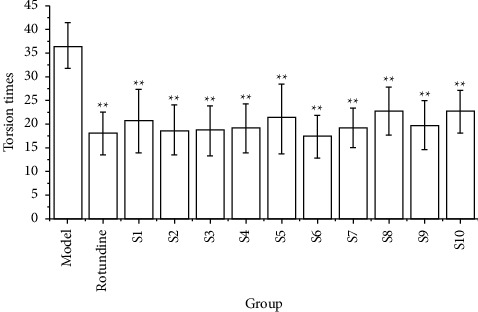
Effects of ten batches of CF aqueous extract on acetic acid-induced torsion response in mice (*n* = 10) (compared with the model group ^*∗*^: *P* < 0.05 and ^*∗∗*^: *P* < 0.01).

In comparison with the model group, the number of torsion was significantly lower (*P* < 0.01) in the CF aqueous extract group of ten different sources, and their analgesic inhibition rates were all greater than 37%, with the inhibition rates in the aqueous extract groups of S2, S3, and S6 being greater than 48%. In comparison with other sources, the analgesic inhibition rate was lower in S8 and S10, with 37.53% and 38.08%, respectively. In comparison with the positive group (rotundine), the analgesic inhibition rate of the CF aqueous extract group was lower than that of the positive group, except for the CF aqueous extract group in S6. At the same time, the experimental results also demonstrated that the ten batches of the CF aqueous extract group would be able to effectively reduce the effect of acetic acid-induced pain in mice, as presented in Table 8 and Figure 7.

### 3.6. Gray Relation Analysis (GRA) between the CF Fingerprint and Its Anti-Inflammatory and Analgesic Activities

The contribution of the common peaks of the CF aqueous extract to the effect of mouse ear swelling can be observed from Table 9 in the order of chromatography: 12 > 3>10 > 7>11 > 5>1 > 4>6 > 2>8 > 9>13, and the correlation of all peaks except peak 13 is greater than 0.6. The contribution of the common peaks of the CF aqueous extract to the effect of mouse torsion can be observed in Table 10 in the order of chromatography: 10 > 4>3 > 9>11 > 8>12 > 7>1 > 5>6 > 2>13, and the correlation of all peaks except peak 13 is greater than 0.6. The results indicated that the anti-inflammatory and analgesic effects of CF were the result of the combined action of multiple compounds. The compounds represented by the common peaks, with the exception of peak 13, were closely related to the anti-inflammatory and analgesic effects of CF.

### 3.7. Partial Least Squares Regression Analysis (PLSR)

#### 3.7.1. PLSR between the CF Fingerprint and Its Anti-Inflammatory Activity

**Figure 8:**
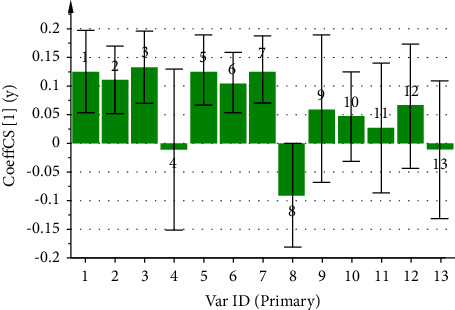
Coefficient plot of partial regression analysis of anti-inflammatory spectrum-effect relationship of CF aqueous extract.

**Figure 9:**
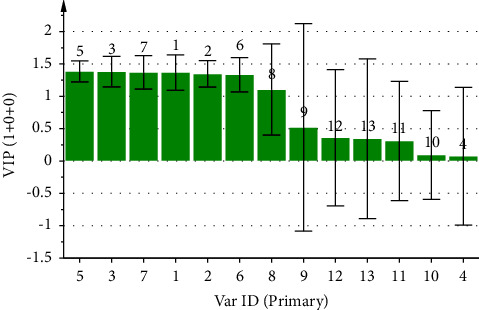
VIP values of common peaks and anti-inflammatory potency of CF aqueous extract.

By utilizing the peak area of each common peak in the fingerprint as the independent variable X and the inhibition rate of the CF aqueous extract on ear swelling in mice as the dependent variable Y, the PLSR analysis of its anti-inflammatory spectrum-effect relationship was performed using the SIMCA 14.1 software, and the regression equation obtained was *Y* = 0.1259X1 + 0.1108X2 + 0.1321X3 − 0.0112X4 + 0.1273X5 + 0.1060X6 + 0.1277X7 − 0.0909X8 + 0.0599X9 + 0.0470X10 + 0.0274X11 + 0.0664X12 − 0.011X13. The values of the model parameters R2X, R2Y, and Q2 are 0.515, 0.704, and 0.595, respectively. In Figure 8, peaks 4, 8, and 13 are negatively correlated with the anti-inflammatory effect, while the rest of the peaks are positively correlated with the anti-inflammatory effect. In Figure 9, the VIP values of peaks 1, 2, 3, 5, 6, and 7 were greater than 1, which indicated that these peaks had a significant effect on the anti-inflammatory effect in mice, among which peak 1 was identified as neochlorogenic acid, peak 2 as chlorogenic acid, and peak 5 as cryptochlorogenic acid, and the anti-inflammatory ability of the CF aqueous extract was significantly enhanced when its content was increased.

#### 3.7.2. PLSR between the CF Fingerprint and Its Analgesic Activity

**Figure 10:**
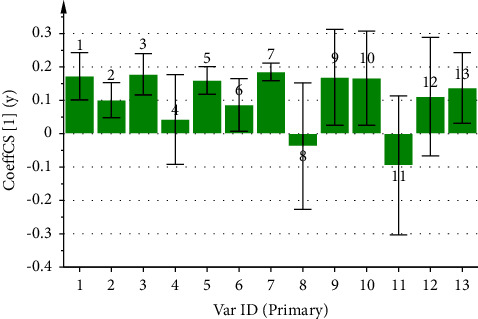
Coefficient plot of partial regression analysis of analgesic spectrum-effect relationship of CF aqueous extract.

**Figure 11:**
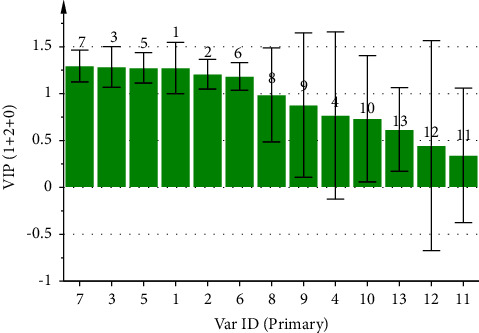
VIP values of common peaks and analgesic potency of CF aqueous extract.

By using the peak area of each common peak in the fingerprint as the independent variable *X* and the analgesic inhibition rate of the CF aqueous extract in mice as the dependent variable *Y*, the regression equation obtained from the PLSR analysis of the analgesic spectrum-effect relationship by utilizing the SIMCA 14.1 software can be represented by *Y* = 0.1629X1 + 0.1104X2 + 0.1654X3 − 0.0885X4 + 0.1538X5 + 0.0985X6 + 0.175X7 − 0.0437X8 + 0.1758X9 + 0.0715X10 − 0.0657X11 + 0.1001X12 + 0.1123X13. The values of the model parameters R2X, R2Y, and Q2 are 0.761, 0.987, and 0.940, respectively. As presented in Figure 10, peaks 8 and 11 are negatively correlated with analgesic effects, while the rest of the peaks are positively correlated with analgesic effects. In Figure 11, the VIP values of peaks 1, 2, 3, 5, 6, and 7 are all greater than 1, indicating that these peaks had a more significant effect on the analgesic efficacy in mice, among which peak 1 was identified as neochlorogenic acid, peak 2 as chlorogenic acid, and peak 5 as cryptochlorogenic acid, and the analgesic ability of the CF aqueous extract was also significantly enhanced when its content was increased.

## 4. Discussion

In the fingerprint section, various columns, wavelengths, flow rates, temperatures, and mobile phases were examined, and the chromatographic conditions that could most comprehensively characterize the information of CF samples were selected in order to produce the HPLC fingerprint with rich chemical information [28]. The common peaks of ten batches of CF were identified at the same time. A total of four components were identified, with peak 1 being neochlorogenic acid, peak 3 being chlorogenic acid, peak 5 being cryptochlorogenic acid, and peak 10 being rosmarinic acid. The results of similarity analysis presented that the fingerprint profiles of ten batches of CF were compared with the reference profiles, and the similarity calculation results were above 0.928, indicating that there were differences in the chemical composition contents between CF aqueous extracts, which might be due to the differences in origin, season, climate, harvesting time, growth condition, and maturity between samples. The results of both CA and PCA indicated that all the ten batches of CF samples were classified into three categories with S1, S3, S5, S7, and S9 as one category, S8 and S10 as one category, and S2, S4, and S6 as one category. Hence, this clearly indicates that the method can be utilized for the quality evaluation of CF samples.

In the anti-inflammatory spectrum-effect relationship study, the results of GRA presented that the correlations of the common peaks contributing to the anti-inflammatory effect were ranked as 12 > 3> 10 > 7> 11 > 5>1 > 4>6 > 2>8 > 9> 13, where except for peak 13, the correlations of all the peaks were greater than 0.6. The PLSR also presented that peaks 4, 8, and 13 were negatively correlated with the anti-inflammatory effect, while the rest of the peaks were positively correlated with the anti-inflammatory effect. The VIP values were ranked as 5 > 3> 7 > 1> 2 > 6>8 > 9> 12 > 13> 11 > 10> 4, whereas the VIP values of peaks 1, 2, 3, 5, 6, and 7 were all greater than 1.

In the study of the analgesic spectrum-effect relationship, the results of the GRA presented that the correlation of the common peaks that contributes to analgesia was in the order of 10 > 4> 3 > 9> 11 > 8> 12 > 7> 1 > 5> 6 > 2> 13, and the correlation of all peaks was greater than 0.5. The PLSR also presented that peaks 8 and 11 were negatively correlated with analgesia, and the rest of the peaks were positively correlated with analgesia. The VIP values of the peaks were ranked as 7 > 3> 5 > 1 > 2 > 6> 8 > 9> 4 > 10 > 13 > 12> 11, among which the VIP values of peaks 1, 2, 3, 5, 6, and 7 were all greater than 1.

By combining the above two analytical methods, the anti-inflammatory and analgesic effects of CF may be the consequence of the combined action of several components. Peaks 1, 2, 3, 5, 6, and 7 may be the basis of the anti-inflammatory and analgesic substances of CF. Among them, peak 1 is neochlorogenic acid, peak 2 is chlorogenic acid, and peak 5 is cryptochlorogenic acid. All of these have been reported to possess satisfactory anti-inflammatory and analgesic activities in the relevant literature [29–31]. In addition, the results of this data processing indicated that the VIP value of peak 10 (rosmarinic acid) was not greater than 1, but the literature has shown that rosmarinic acid is an active anti-inflammatory and analgesic component in herbs and is also one of the quality control indicators of *Sarcandrae Herba* of the CF family plants [32, 33]. As such, rosmarinic acid should also be the CF anti-inflammatory and analgesic potent substance. Lastly, the other chromatographic peaks were not successfully identified, and further in-depth studies are required in the future.

## 5. Conclusion

CF is a widely used medicinal plant employed by the Yao ethnic group in China. It has been successfully utilized in clinical settings to treat several inflammatory disorders, demonstrating the herb's potent anti-inflammatory and analgesic effects. In this study, the fingerprints of ten batches of CF from different sources were established, and a total of 13 common peaks were chosen, among which 4 components were identified. The results of similarity analysis displayed that the similarity between the fingerprint profiles of each origin and the reference profile (*R*) was greater than 0.900. Hence, this indicates that the preparation method of CF was stable and that the deviation between the origins was relatively small, which was in line with the requirements of fingerprinting research. Simultaneously, the anti-inflammatory and analgesic activities of CF were also comprehensively evaluated with the xylene ear swelling test and acetic acid torsion test, and the results presented that CF possesses satisfactory anti-inflammatory and analgesic activities. The anti-inflammatory and analgesic spectrum-effect relationship of CF was established by combined GRA and PLSR, whereby it was revealed that neochlorogenic acid, chlorogenic acid, cryptochlorogenic acid, and rosmarinic acid may be the anti-inflammatory and analgesic potent substances of CF. As such, this study acts as a basis and provides a scientific basis for the establishment of CF quality standards as well as their further development and utilization.

## Figures and Tables

**Table 1:** Source information of CF samples.

Sample number	Sources	Collection date
S1	Jinxiu county, Laibin city, Guangxi, China	2021.04.07
S2	Yuzhou district, Yulin city, Guangxi, China	2021.04.23
S3	Xiangzhou county, Laibin city, Guangxi, China	2021.05.13
S4	Qintang district, Guigang city, Guangxi, China	2021.05.07
S5	Shanglin county, Nanning city, Guangxi, China	2021.05.12
S6	Babu district, Hezhou city, Guangxi, China	2021.05.26
S7	Luzhai county, Liuzhou city, Guangxi, China	2021.04.18
S8	Longsheng county, Guilin city, Guangxi, China	2021.05.14
S9	Wuming district, Nanning city, Guangxi, China	2021.05.21
S10	Gongcheng county, Guilin city, Guangxi, China	2021.04.15

**Table 2:** The relative retention time of the common peaks of 10 batches of CF samples.

Peak/no.	S1	S2	S3	S4	S5	S6	S7	S8	S9	S10	RSD (%)
1	0.3607	0.3593	0.3595	0.3585	0.3599	0.3580	0.3596	0.3600	0.3595	0.3607	0.23
2	0.4502	0.4505	0.4506	0.4499	0.4497	0.4493	0.4504	0.4504	0.4487	0.4502	0.14
3	0.4622	0.4609	0.4611	0.4608	0.4614	0.4602	0.4617	0.4621	0.4609	0.4622	0.15
4	0.4773	0.4854	0.4851	0.4853	0.4850	0.4852	0.4857	0.4859	0.4847	0.4856	0.53
5	0.4927	0.5013	0.5019	0.5008	0.5028	0.4999	0.5025	0.5031	0.5024	0.5040	0.64
6	0.6203	0.6198	0.6198	0.6195	0.6196	0.6176	0.6203	0.6194	0.6174	0.6203	0.17
7	0.6457	0.6449	0.6445	0.6450	0.6451	0.6428	0.6459	0.6448	0.6427	0.6457	0.17
8	0.9092	0.9094	0.9091	0.9086	0.9082	0.9088	0.9093	0.9087	0.9084	0.9092	0.04
9	0.9383	0.9381	0.9371	0.9371	0.9363	0.9369	0.9380	0.9368	0.9363	0.9383	0.08
10 (S)	1.0000	1.0000	1.0000	1.0000	1.0000	1.0000	1.0000	1.0000	1.0000	1.0000	0.00
11	1.0533	1.0537	1.0534	1.0526	1.0520	1.0530	1.0534	1.0525	1.0525	1.0533	0.05
12	1.2314	1.2321	1.2322	1.2306	1.2303	1.2313	1.2317	1.2305	1.2307	1.2314	0.06
13	1.2684	1.2691	1.2692	1.2675	1.2671	1.2685	1.2685	1.2675	1.2680	1.2684	0.05

**Table 3:** The relative peak areas of the common peaks of 10 batches of CF samples.

Peak/no.	S1	S2	S3	S4	S5	S6	S7	S8	S9	S10	RSD (%)
1	0.6628	0.9304	0.7619	1.2910	0.6998	1.2493	0.6325	0.312	0.6326	0.3426	43.69
2	0.0405	0.0585	0.0448	0.0901	0.0404	0.0701	0.0356	0.0203	0.0314	0.0183	49.6
3	0.7373	0.9969	0.8395	1.2500	0.8047	1.2547	0.6862	0.3957	0.6744	0.4186	36.78
4	0.0776	0.0515	0.0825	0.1059	0.1717	0.0928	0.0606	0.0558	0.1143	0.0645	41.35
5	0.5620	0.7679	0.6599	1.0552	0.5925	0.9689	0.5210	0.2680	0.4965	0.2878	41.71
6	0.0690	0.0991	0.0712	0.1508	0.0638	0.1095	0.0690	0.0330	0.0445	0.0283	50.65
7	0.0757	0.1043	0.0830	0.1254	0.0805	0.1280	0.0744	0.0307	0.0695	0.0260	42.76
8	0.3890	0.3198	0.3866	0.5437	0.7315	0.4673	0.4497	0.5508	0.4840	0.5057	23.58
9	1.3256	1.0232	1.4339	1.4255	2.0323	1.4888	1.0265	0.6241	1.6550	0.8694	31.9
10 (S)	1.0000	1.0000	1.0000	1.0000	1.0000	1.0000	1.0000	1.0000	1.0000	1.0000	0.00
11	0.2346	0.2618	0.2848	0.3907	0.3882	0.2477	0.2626	0.2752	0.1858	0.2532	23.13
12	0.1360	0.1332	0.1816	0.2107	0.2606	0.2063	0.1767	0.1894	0.1160	0.1211	26.85
13	0.0820	0.0476	0.0828	0.1227	0.2014	0.1738	0.1460	0.1519	0.1175	0.0876	39.17

**Table 4:** Similarity evaluation results of 10 batches of CF samples.

	S1	S2	S3	S4	S5	S6	S7	S8	S9	S10
S1	1	0.970	0.998	0.964	0.977	0.97	0.991	0.919	0.99	0.961
S2	0.97	1	0.976	0.991	0.91	0.991	0.976	0.884	0.929	0.908
S3	0.998	0.976	1	0.974	0.976	0.979	0.989	0.904	0.986	0.947
S4	0.964	0.991	0.974	1	0.923	0.997	0.966	0.858	0.929	0.887
S5	0.977	0.91	0.976	0.923	1	0.931	0.958	0.89	0.992	0.941
S6	0.97	0.991	0.979	0.997	0.931	1	0.968	0.854	0.941	0.889
S7	0.991	0.976	0.989	0.966	0.958	0.968	1	0.953	0.97	0.974
S8	0.919	0.884	0.904	0.858	0.89	0.854	0.953	1	0.899	0.987
S9	0.99	0.929	0.986	0.929	0.992	0.941	0.97	0.899	1	0.953
S10	0.961	0.908	0.947	0.887	0.941	0.889	0.974	0.987	0.953	1
R	0.997	0.979	0.997	0.976	0.972	0.979	0.996	0.928	0.981	0.961

**Table 5:** Principal component eigenvalues and variance contribution rate.

Peak/no.	Eigenvalue	Variance contribution rate (%)	Cumulative variance contribution rate (%)
1	6.75	51.93	51.93
2	2.21	16.98	68.91
3	1.88	14.49	83.40
4	0.99	7.58	90.98
5	0.85	6.54	97.52
6	0.26	2.03	99.56
7	0.04	0.32	99.88
8	0.01	0.11	99.99
9	0.00	0.01	100.00
10	0.00	0.00	100.00
11	0.00	0.00	100.00
12	0.00	0.00	100.00
13	0.00	0.00	100.00

**Table 6:** Principal component analysis loading matrix.

Peak	Principal component		
no.	1	2	3
1	0.982	0.089	0.03
2	0.981	−0.039	0.025
3	0.986	0.066	0.007
4	−0.057	0.93	0.177
5	0.994	0.051	0.027
6	0.968	−0.14	0.07
7	0.976	0.119	0.038
8	−0.818	0.119	0.404
9	0.349	0.814	−0.031
10	0.02	−0.375	−0.355
11	0.214	−0.579	0.444
12	0.214	−0.161	0.908
13	−0.29	0.26	0.762

**Table 7:** Effects of ten batches of CF aqueous extract on xylene-induced ear swelling in mice (*n* = 10).

Groups	Dosage of administration (g/kg)	Swelling degree (*x* ± *s*, mg)	Inhibition ratio (%)
Model	—	5.45 ± 1.16	0.00
Positivity (dexamethasone)	0.006	2.07 ± 0.74^*∗∗*^	62.02
S1	7.28	3.24 ± 0.58^*∗∗*^	40.55
S2	7.28	2.52 ± 0.71^*∗∗*^	53.76
S3	7.28	2.25 ± 1.02^*∗∗*^	58.72
S4	7.28	3.10 ± 0.85^*∗∗*^	43.12
S5	7.28	3.37 ± 0.86^*∗∗*^	38.17
S6	7.28	2.20 ± 0.80^*∗∗*^	59.63
S7	7.28	2.84 ± 0.83^*∗∗*^	47.89
S8	7.28	3.50 ± 0.93^*∗∗*^	35.78
S9	7.28	3.19 ± 1.35^*∗∗*^	41.47
S10	7.28	3.42 ± 0.86^*∗∗*^	37.25

Compared with the model group ^*∗*^: *P* < 0.05 and ^*∗∗*^: *P* < 0.01.

**Table 8:** Effects of ten batches of CF aqueous extract on acetic acid-induced torsion response in mice (*n* = 10).

Group	Dosage of administration (g/kg)	Torsion times (*x* ± *s*, time)	Inhibition ratio (%)
Model	—	36.5 ± 4.86	0.00
Positive (rotundine)	0.05	17.9 ± 4.61^*∗∗*^	50.96
S1	7.28	20.5 ± 6.62^*∗∗*^	43.84
S2	7.28	18.7 ± 5.27^*∗∗*^	48.77
S3	7.28	18.6 ± 5.28^*∗∗*^	49.04
S4	7.28	19.2 ± 5.20^*∗∗*^	47.40
S5	7.28	21.2 ± 7.36^*∗∗*^	41.92
S6	7.28	17.3 ± 4.64^*∗∗*^	52.60
S7	7.28	19.3 ± 4.35^*∗∗*^	47.12
S8	7.28	22.8 ± 5.07^*∗∗*^	37.53
S9	7.28	19.8 ± 5.22^*∗∗*^	45.75
S10	7.28	22.6 ± 4.55^*∗∗*^	38.08

Compared with the model group ^*∗*^: *P* < 0.05 and ^*∗∗*^: *P* < 0.01.

**Table 9:** Correlation between the inhibition rate of mouse ear swelling and common peak area.

Peak/no.	Correlation
1	0.6865
2	0.6450
3	0.7317
4	0.6470
5	0.7028
6	0.6470
7	0.7155
8	0.6439
9	0.6261
10	0.7280
11	0.7055
12	0.7350
13	0.5436

**Table 10:** Correlation between the analgesic inhibition rate and common peak area.

Peak/no.	Correlation
1	0.7024
2	0.6640
3	0.7353
4	0.7493
5	0.7002
6	0.6717
7	0.7174
8	0.7245
9	0.7350
10	0.8058
11	0.7328
12	0.7224
13	0.5974

## Data Availability

The data used to support the findings of this study are included within the article.

## References

[1] Editorial Committee of ’Chinese Materia Medica’ of State Administration of Traditional Chinese Medicine (1999). *Chinese Materia Medica*.

[2] Dai B. (2009). *Chinese Modern Yao Medicine*.

[3] Chen F., Zou Y., Chen J. (2020). Study on chemical constituents of Chloranthus spicatus. *Chinese Herbal Medicine*.

[4] Chen Y., Yang L., Huang W. (2021). FBXL19-AS1 aggravates the progression of hepatocellular cancer by downregulating KLF2. *Research and development of natural products*.

[5] Kay J., Thadhani E., Samson L., Engelward B. (2019). Inflammation-induced DNA damage, mutations and cancer. *DNA Repair*.

[6] Roe K. (2021). An inflammation classification system using cytokine parameters. *Scandinavian Journal of Immunology*.

[7] Fredman G., MacNamara K. C. (2021). Atherosclerosis is a major human killer and non-resolving inflammation is a prime suspect. *Cardiovascular Research*.

[8] Makris U. E., Abrams R. C., Gurland B., Reid M. C. (2014). Management of persistent pain in the older patient: a clinical review. *JAMA*.

[9] Qaseem A., Wilt T. J., McLean R. M., Forciea M. A. (2017). Noninvasive treatments for acute, subacute, and chronic low back pain: a clinical practice guideline from the American college of physicians. *Annals of Internal Medicine*.

[10] Taylor A. M., Phillips K., Patel K. V. (2016). Assessment of physical function and participation in chronic pain clinical trials: IMMPACT/OMERACT recommendations. *Pain*.

[11] Matsuda M., Huh Y., Ji R. R. (2019). Roles of inflammation, neurogenic inflammation, and neuroinflammation in pain. *Journal of Anesthesia*.

[12] Xiong H. Y., Zhang Z. J., Wang X. Q. (2021). Bibliometric analysis of research on the comorbidity of pain and inflammation. *Pain Research and Management*.

[13] Seifert O., Baerwald C. (2021). Interaction of pain and chronic inflammation. *Zeitschrift für Rheumatologie*.

[14] Bindu S., Mazumder S., Bandyopadhyay U. (2020). Non-steroidal anti-inflammatory drugs (NSAIDs) and organ damage: a current perspective. *Biochemical Pharmacology*.

[15] Ribeiro H., Rodrigues I., Napoleão L. (2022). Non-steroidal anti-inflammatory drugs (NSAIDs), pain and aging: adjusting prescription to patient features. *Biomedicine & Pharmacotherapy*.

[16] Tai F. W. D., McAlindon M. E. (2021). Non-steroidal anti-inflammatory drugs and the gastrointestinal tract. *Clinical Medicine*.

[17] Ishitsuka Y., Kondo Y., Kadowaki D. (2020). Toxicological property of acetaminophen: the dark side of a safe antipyretic/analgesic drug. *Biological and Pharmaceutical Bulletin*.

[18] Lan L., Sun W., Chang Q., Sun G. (2021). Comprehensive evaluation of Licorice extract by five-dimensional quantitative profiling. *Journal of Chromatography A*.

[19] Bao H., Yang H., Li J., Xu Y., Huang X. (2022). Establishment and development of a quality evaluation method for sangbaipi decoction. *Journal of AOAC International*.

[20] Zhang K., Shen X., Yang L. (2022). Exploring the Q-markers of Angelica sinensis (Oliv.) Diels of anti-platelet aggregation activity based on spectrum-effect relationships. *Biomedical Chromatography: Biomedical Chromatography*.

[21] Fan Q., Yang R., Yang F., Xia P., Zhao L. (2020). Spectrum-effect relationship between HPLC fingerprints and antioxidant activity of Angelica sinensis. *Biomedical Chromatography: Biomedical Chromatography*.

[22] Qiu Q., Shi J., Huang C. (2022). Spectrum-effect relationship between hplc fingerprint and anti-inflammatory activity of n-butanol parts of Tetrastigma planicaule (Hook) Gagnep. *Pakistan journal of pharmaceutical sciences*.

[23] Wang F., Qian Z., Liao G. (2022). HPLC coupled with chemical fingerprinting for multi-pattern recognition for identifying the authenticity of clematidis armandii caulis. *Journal of Visualized Experiments: JoVE*.

[24] Hu N., Wang C., Wang B. (2022). Qianghuo Shengshi decoction exerts anti-inflammatory and analgesic via MAPKs/CREB signaling pathway. *Journal of Ethnopharmacology*.

[25] Dai G., Li B., Xu Y., Li Z., Mo F., Wei C. (2021). Synergistic interaction between matrine and paracetamol in the acetic acid writhing test in mice. *European Journal of Pharmacology*.

[26] Qiu Q., Jiang L., Huang C. (2022). Study on the spectrum-effect correlation of anti-inflammatory active extract of Sauropus spatulifolius beille. *Journal of Analytical Methods in Chemistry*.

[27] Wang L. J., Jiang Z. M., Xiao P. T., Sun J. B., Bi Z. M., Liu E. H. (2019). Identification of anti-inflammatory components in Sinomenii Caulis based on spectrum-effect relationship and chemometric methods. *Journal of Pharmaceutical and Biomedical Analysis*.

[28] Zhu Z. F., Fan Q. M., Liu Y. Z. (2020). The UPLC fingerprint of Shentong Zhuyu Decoction was optimized based on the principle of information entropy maximization. *Chinese herbal medicine*.

[29] Kim M., Choi S. Y., Lee P., Hur J. (2015). Neochlorogenic acid inhibits lipopolysaccharide-induced activation and pro-inflammatory responses in BV2 microglial cells. *Neurochemical Research*.

[30] Bagdas D., Gul Z., Meade J. A., Cam B., Cinkilic N., Gurun M. S. (2020). Pharmacologic overview of chlorogenic acid and its metabolites in chronic pain and inflammation. *Current Neuropharmacology*.

[31] Zhao X. L., Yu L., Zhang S. D. (2020). Cryptochlorogenic acid attenuates LPS-induced inflammatory response and oxidative stress via upregulation of the Nrf2/HO-1 signaling pathway in RAW 264.7 macrophages. *International Immunopharmacology*.

[32] Zhou H., Liang J., Lv D. (2013). Characterization of phenolics of Sarcandra glabra by non-targeted high-performance liquid chromatography fingerprinting and following targeted electrospray ionisation tandem mass spectrometry/time-of-flight mass spectrometry analyses. *Food Chemistry*.

[33] Dahchour A. (2022). Anxiolytic and antidepressive potentials of rosmarinic acid: a review with a focus on antioxidant and anti-inflammatory effects. *Pharmacological Research*.

